# Functional changes in hemostasis during asexual and sexual parasitemia in a controlled human malaria infection

**DOI:** 10.1371/journal.pone.0271527

**Published:** 2022-07-15

**Authors:** Shengshi Huang, Wouter van der Heijden, Isaie J. Reuling, Jun Wan, Qiuting Yan, Romy M. W. de Laat - Kremers, Andre J. Van der Ven, Philip G. de Groot, Matthew McCall, Robert W. Sauerwein, Teun Bousema, Mark Roest, Marisa Ninivaggi, Quirijn de Mast, Bas de Laat

**Affiliations:** 1 Department of Functional Coagulation, Synapse Research Institute, Maastricht, The Netherlands; 2 Department of Biochemistry, Cardiovascular Research Institute Maastricht, Maastricht University, Maastricht, The Netherlands; 3 Department of Internal Medicine, Radboud Center for Infectious Diseases, Radboud University Medical Centre, Nijmegen, The Netherlands; 4 Department of Medical Microbiology, Radboud University Medical Centre, Nijmegen, The Netherlands; 5 Department of Data Analysis and Artificial Intelligence, Synapse Research Institute, Maastricht, The Netherlands; 6 Department of Platelet pathophysiology, Synapse Research Institute, Maastricht, The Netherlands; King Faisal University, SAUDI ARABIA

## Abstract

Decreased platelet count is an early phenomenon in asexual *Plasmodium falciparum* parasitemia, but its association with acute or long-term functional changes in platelets and coagulation is unknown. Moreover, the impact of gametocytemia on platelets and coagulation remains unclear. We investigated the changes in platelet number and function during early asexual parasitemia, gametocytemia and convalescence in 16 individuals participating in a controlled human malaria infection study, and studied its relationship with changes in total and active von Willebrand factor levels (VWF) and the coagulation system. Platelet activation and reactivity were determined by flow cytometry, and the coagulation system was assessed using different representative assays including antigen assays, activity assays and global functional assays. Platelet count was decreased during asexual blood stage infection but normalized during gametocytemia. Platelet P-selectin expression was slightly increased during asexual parasitemia, gametocytemia and at day 64. In contrast, platelet reactivity to different agonists remained unchanged, except a marked decrease in reactivity to low dose collagen-related peptide-XL. Thrombin generation and antigen assays did not show a clear activation of the coagulation during asexual parasitemia, whereas total and active VWF levels were markedly increased. During gametocytemia and on day 64, the endogenous thrombin potential, thrombin peak and velocity index were increased and prothrombin conversion and plasma prothrombin levels were decreased. We conclude that the decreased platelet count during asexual parasitemia is associated with increased active VWF levels (i.e. endothelial activation), but not platelet hyperreactivity or hypercoagulability, and that the increased platelet clearance in asexual parasitemia could cause spontaneous VWF-platelet complexes formation.

## Introduction

Malaria is the most important parasitic disease worldwide, leading to approximately 627,000 deaths in 2020 [1]. *Plasmodium falciparum* is responsible for by far the most deaths and severe illness. Thrombocytopenia is a common and consistent feature of malaria. Thrombocytopenia occurs early during asexual parasitemia, but whether this reduction of platelet number is associated with changes in the function of platelets is uncertain. Besides their well-known roles in hemostasis and preservation of vascular integrity, platelets are increasingly recognized for their roles in immunity and host defense. Interestingly, activated platelets were shown to kill malaria parasites inside red blood cells [2, 3]. The function of platelets can be assessed by determining their reactivity to *ex vivo* stimulation by different platelet agonists. Moreover, the mechanisms that are responsible for the early changes in platelets are incompletely understood. Previously reported mechanisms included activation of platelets and the endothelium, leading to the release of Von Willebrand factor (VWF) from the Weibel-Palade bodies in the endothelium, as well as the activation of the coagulation system [4–6]. Released VWF becomes activated in the circulation, in order to capture platelets. The A1 domain of VWF requires a conformational change to convert into its active conformation [7]. We have developed an enzyme-linked immunosorbent assay for the detection of the VWF A1 domain in an “open conformation”, also published as “active VWF” [8]. In addition, thrombin generation (TG) assays can be used for a global assessment of the coagulation system. The coagulation cascade provides a second link to the immune system, as key coagulation enzyme thrombin plays a important role in inflammatory processes and the inflammation-coagulation axis [9]. Thrombin cleaves fibrinogen to fibrin and activates both platelets and endothelial cells via proteolytic cleavage of protease-activated receptors (PARs). To maintain the hemostatic balance, excessive thrombin must be timely inactivated by anticoagulant factors, such as antithrombin and α_2_Macroglobulin (α_2_M). Antithrombin is produced in the liver and is responsible for ~80% of the anticoagulant activity whereas α_2_M is responsible for ~10%. TG curves can be split into prothrombin conversion and thrombin inactivation, using the computational thrombin dynamics method, as previously described by Kremers et al. [10–12].

Another unresolved question is whether functional changes in platelets and coagulation are an acute phenomenon or may persist for a longer time. Recently, we showed that a controlled human malaria infection (CHMI) induces long-term functional changes in monocytes [13]. After clearance of asexual blood-stage parasites, sexual stage parasites (gametocytes) may emerge from the bone marrow. Gametocytes circulate at low densities and do not cause clinical symptoms. Immature gametocytes home to the bone marrow and spleen, crossing the endothelial barrier, and upon maturation, intravasate back into the circulation [14]. Gametocytes were shown to stimulate the release of angiogenic factors and induce alterations in the endothelial lining of the bone marrow [15], but whether gametocytes also have systemic effects on platelets or coagulation is unknown.

Controlled human malaria infections (CHMI) offer a unique model to investigate in a detailed and standardized manner the functional changes that occur during early asexual falciparum parasitemia. Previous studies that used samples from CHMI participants have reported changes in platelets, endothelial activation [4, 16] and thrombin generation [17] during asexual parasitemia, but have not directly interrelated these processes, nor have they assessed the duration of these changes. The aim of our present study was to assess changes in the number and function of platelets and the relation with changes in total and active VWF and the coagulation system during early asexual parasitemia, subsequent gametocytemia and at convalescence in blood samples from participants in a CHMI that was designed to induce gametocytemia.

## Material and methods

### Samples

The present study was performed with blood samples from a CHMI (ClinicalTrials.gov, NCT02836002), which was conducted at the Radboud university medical center (Nijmegen, the Netherlands after approval of the local medical ethics board and in accordance with the Declaration of Helsinki. Details and results of the primary objectives of this study have been published earlier [18, 19]. In summary, the CHMI concerned an open-label randomized trial in 16 healthy malaria-naïve participants aged 18–35 years, who were infected with the *P*. *falciparum* strain 3D7 by bites of infected Anopheles mosquitoes. The primary objective of the trial was to safely induce gametocytemia in study participants by the use of different (sub)curative drug regimens based on sulfadoxine-pyrimethamine. Samples from the following four time points were analyzed: 1) BL, baseline prior the challenge; 2) DT1, the day malaria parasites were detected at a density of ≥5000 parasites per milliliter by qPCR or a positive thick blood smear, which was the time of treatment onset with a subcurative dose of sulfadoxine-pyrimethamine or piperaquine phosphate; 3) DT2, 14 days after DT1, which roughly corresponded with carriership of gametocytes; 4) C64, which is 64 days after start of treatment.

### Platelet flow cytometry

Platelet flow cytometry was performed using citrated whole blood (3.2% sodium citrate, Becton Dickinson, Franklin Lakes, NJ, USA) between 1–3 hours after blood collection at the Radboud university medical center (Radboudumc), Nijmegen, the Netherlands, as previously described [20]. In short, platelet activation status as well as platelet reactivity were assessed by quantifying the platelet membrane expression of the α-granule protein P-selectin (CD62P) and the binding of fibrinogen to the activated integrin αIIbβ3 in unstimulated whole blood samples and after *ex vivo* stimulation with a low or high concentration of adenosine diphosphate (ADP, 2.5 μM and 62.5 μM, Sigma-Aldrich, USA), thrombin receptor activation peptide-6 (TRAP-6, 6.25 μM and 25 μM, Sigma-Aldrich, USA) or collagen-related peptide (CRP-XL, 2.5 μg/mL and 62.5 μg/mL). Platelets were stained using anti-CD61 (Beckman Coulter, Brea. CA, USA), anti-P-selectin (Biolegend, San Diego, CA, USA) and anti-fibrinogen (DAKO, Santa Clara, CA) antibodies and fixated in 0.2% paraformaldehyde. Samples were measured on a FC500 flow cytometer (Beckman Coulter, Brea, USA). Data were extracted using Kaluza 2.1 (Beckman Coulter), normalized against quality controls to ensure measurement stability and are expressed as median fluorescence intensity (MFI). Platelet count and mean platelet volume (MPV) were determined using an automated hematology analyzer (Sysmex, Kobe, Japan).

### VWF antigen and activity assays

Determination of total VWF antigen levels was performed with an in-house developed sandwich ELISA assay. In short, polyclonal rabbit anti-VWF antibody (Dako, Glostrup, Denmark) in 100mM carbonate-bicarbonate coating buffer (pH 9.6) was coated overnight in 96 wells microtiter plates at 4°C. After washing the plates, blocking was performed using BSA-containing phosphate-buffered saline (PBS/2% BSA/0.5% Tween-20). This was followed by adding 640-time diluted plasma samples and incubated at room temperature (RT) for 2 hours. After washing the plates were incubated with an HRP-conjugated polyclonal rabbit anti-VWF antibody (Dako, Glostrup, Denmark) for 2 hours at RT. Plates were washed before addition of 3,3′,5,5′-Tetramethylbenzidine (TMB; Sigma-Aldrich, Zwijndrecht, the Netherlands) for 15 minutes at RM. The reaction was stopped with 2 M sulfuric acid (H_2_SO_4_; Sigma-Aldrich, Zwijndrecht, the Netherlands). Optical densities (OD) were measured at 450 nm using an ELx808 Absorbance Microplate Reader (Biotek, Bad Friedrichshall, Germany). Results were normalized (%) by normal pool plasma (NPP) which was present as a standard in every plate.

The assay for measuring active VWF has previously been described in detail [21]. In short, 96 wells microtiter plates (NUNC Maxisorp, Thermo Fisher Scientific, Waltham, USA) were coated with 1.98 μg/ml VHH against active VWF in a carbonate-bicarbonate coating buffer (pH 9.6). After washing 5 times with washing buffer (0.01% Tween-20 in phosphate-buffered saline (PBS)), the plates were blocked by 2% BSA in PBS for 50 minutes at RT. After washing 5 times with washing buffer, 20 times diluted plasma samples by PBS/1% BSA were incubated for 2 hours at RT. Washing plates and incubating with HRP-conjugated anti-VWF antibodies (1.2 μg/mL) in PBS/1% BSA for 2 hours at RT. Washing plates and incubating with SIGMAFAST OPD (Sigma) for 15 minutes. 2 M sulfuric acid (H_2_SO_4_, Sigma) was used to terminate the reaction and optical densities (OD) were measured at 490 nm using an ELx808 Absorbance Microplate Reader (Biotek, Bad Friedrichshall, Germany). Results were normalized (%) compared to normal pool plasma (NPP) as a standard in every plate.

### Coagulation factor antigen and activity assays

The determination of prothrombin levels was performed with an in-house developed sandwich ELISA assay. In short, affinity-purified sheep anti-human prothrombin IgG antibody (Affinity Biologicals, Ancaster, Canada) in 100mM carbonate-bicarbonate coating buffer (pH 9.6) was coated overnight in 96 wells microtiter plates at 4°C. After washing the plates, blocking was performed using phosphate-buffered saline (PBS/2% BSA/0.5% Tween-20) for 45 minutes at RT. This was followed by adding 1280-time diluted plasma samples, incubation was performed at RT for 2 hours. After washing the plates, the plates were incubated with a HRP-conjugated sheep anti-human prothrombin IgG antibody (Affinity Biologicals, Ancaster, Canada) in PBS/1% BSA/0.5% Tween-20 for 2 hours at RT. Plates were washed before addition of SIGMAFAST o-phenylenediamine dihydrochloride (OPD) for 15 minutes at RM. The reaction was stopped adding 2 M sulfuric acid (H_2_SO_4_). Optical density (OD) was measured at 490 nm using an ELx808 Absorbance Microplate Reader. Results were normalized (%) compared to normal pool plasma (NPP) as a standard in every plate.

Determination of protein S levels was performed with an in-house developed sandwich ELISA assay. In short, Affinity-purified sheep protein S (PS) IgG antibody (Affinity Biologicals, Ancaster, Canada) was coated overnight in 96 wells microtiter plates at 4°C in coating buffer. After washing the plates, blocking was performed using phosphate-buffered saline (PBS/2% BSA/0.5% Tween-20), followed by adding 200-time diluted plasma samples to the plate. After incubation and washing, the plates were incubated with an HRP-conjugated sheep anti-human PS IgG antibody (Affinity Biologicals, Ancaster, Canada) for detection. After incubation, the plates were washed before adding 3,3′,5,5′-Tetramethylbenzidine (TMB) for ~15 minutes at RM. The reaction was stopped with 2 M sulfuric acid (H2SO4). Optical density (OD) was measured at 450 nm using an ELx808 Absorbance Microplate Reader. Results were normalized (%) compared to normal pool plasma (NPP) as a standard in every plate.

Antithrombin (AT), α2M and fibrinogen levels were measured to serve as input data for the thrombin dynamics analysis. Antithrombin was measured on the automated coagulation analyzer STA-R (Diagnostica Stago, Asnières sur Seine, France), using the STA Stachrom AT III reagent according to manufacturer specifications. Plasma α_2_M levels were detected by an in-house chromogenic assay as previously described by Kremers et al. [10]. Fibrinogen was measured using the STA Fibrinogen reagent on the STart (Diagnostica Stago, Asnières sur Seine, France) as previously described [10].

### Thrombin generation

Thrombin generation (TG) was assessed using the calibrated automated thrombogram (CAT) method which is previously described in detail [22]. In short, TG was triggered using PPP reagent (Diagnostica Stago, Asnières, France), in the presence or absence of 7 nM recombinant human thrombomodulin (TM) (made in-house). This concentration of thrombomodulin was used as it caused 50% inhibition of the ETP in healthy subjects. All wells contained 80 ul of plasma with either 20 μL TF/phospholipids(± TM) or 20 μL calibrator. TG was triggered by the automated addition of FluCa Diagnostica Stago, Asnières sur Seine, France, containing 2.5 mM ZGGR-AMC and 100 mM CaCl_2_. Fluorescence data was recorded and converted using dedicated software (Diagnostica Stago, Asnières sur Seine, France). TG data was normalized using normal pool plasma (NPP) measured as a standard in every plate.

### Thrombin dynamics

Thrombin dynamics analysis was used to calculate prothrombin conversion and thrombin inactivation parameters from each TG curve. Thrombin inactivation was predicted by a previously described and validated computational model [10, 23–25]. This model consists of a set of ordinary differential equations, which describe the rate of thrombin inactivation in time based on the plasma AT, α_2_M and fibrinogen level and the free thrombin concentration at each point in time (Eq 1–3).


d(T‐AT)/dt=kAT⋅[AT]t⋅[Tfree]t Eq 1

$$d(T‐\alpha_{2}M)/dt=k\alpha_{2}M\cdot [\alpha_{2}M]t\cdot [Tfree]t$$ Eq 2

$$‐d(Tfree)/dt=kAT\cdot [AT]t\cdot [Tfree]t+k\alpha_{2}M\cdot [\alpha_{2}M]t\cdot [Tfree]t$$ Eq 3

The amount of available thrombin (Tfree) depends on the amount of thrombin substrate ZGGR-AMC that is present, and rate constants for the inactivation of thrombin by antithrombin (kAT) and α_2_-macroglobulin (kα_2_M), which are dependent on the plasma fibrinogen level, as described in more detail elsewhere [10].

During the course of the TG process, at each time point, the TG curve is the net result of prothrombin conversion and thrombin inactivation. Therefore, the course of prothrombin conversion (d(P)/dt) can be calculated from the TG curve ([T]t) and the inactivation rate of thrombin at a specific thrombin concentration (d(T-inh)/dt) (Eq 4). With the previously described model for thrombin inactivation we can calculate the thrombin inactivation rate at each time point during TG (Eq 5).


d(T)/dt=‐d(P)/dt‐d(T‐inh)/dt Eq 4

$$‐d(P)/dt=d(T)/dt+kAT\cdot [AT]t\cdot [T]t+k\alpha_{2}M\cdot [\alpha_{2}M]t\cdot [T]t$$ Eq 5

Thrombin inactivation can be quantified independent of the plasma prothrombin level by computing the thrombin decay capacity. This the pseudo-first order decay constant for thrombin that combines the overall effect of thrombin inactivation by AT and α_2_M, and it translates into the capacity of a plasma sample to inhibit thrombin.

The prothrombin conversion curve described the rate of prothrombin activation at each time point during TG. It is quantified by its area-under-the-curve, which translates to the total amount of prothrombin converted (PC_tot_) throughout the TG experiment, and the peak height of the prothrombin conversion curve, which is the maximum prothrombin conversion rate (i.e. the maximum activity of the prothrombinase complex; PC_max_).

### Statistics

Statistical analysis was performed using GraphPad Prism software (Prism version 8.0.1 for Windows). Group variables were described by absolute figures and percentages. Statistical differences between groups were assessed by using repeated measures ANOVA. Results were expressed as the median with the interquartile range (IQR). Distribution of continuous data was assessed by Shapiro-Wilks test and correlations were described by Spearman’s rank correlation coefficient. Differences between values were considered when *P <* 0.05.

### Data sharing statement

For original data, please contact r.delaat@thrombin.com.

## Results

### Platelet reactivity

**Fig 1:**
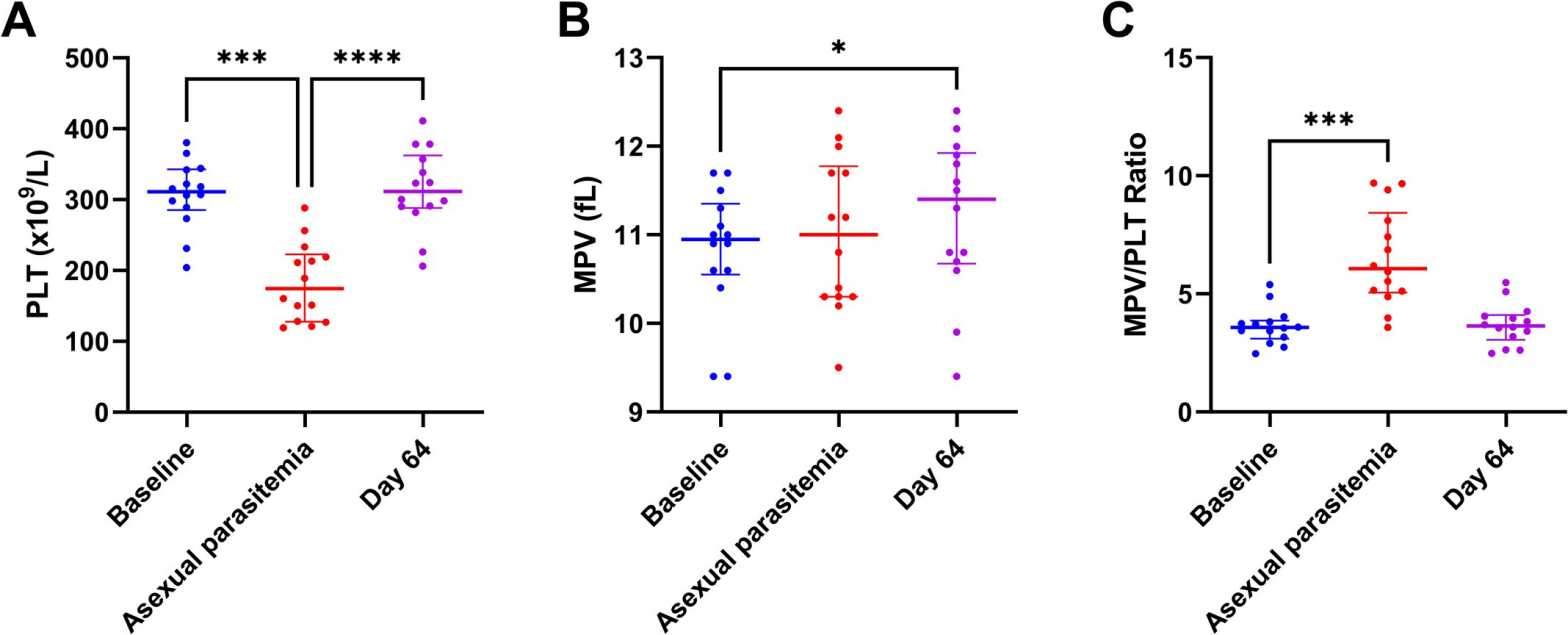
Platelet count and volume in the three stages of *plasmodium falciparum* infection. (A) The platelet count is significantly reduced during asexual parasitemia compared to the platelet count at baseline and at convalescence (day 64). (B) The mean platelet volume (MPV) is increased during convalescence compared to the baseline level. (C) The MPV/platelet ratio is increased during asexual parasitemia compared to baseline. Results are shown as the median and interquartile range, and *, *** and **** indicate p-values below 0.05, 0.001 and 0.0001, respectively.

**Fig 2:**
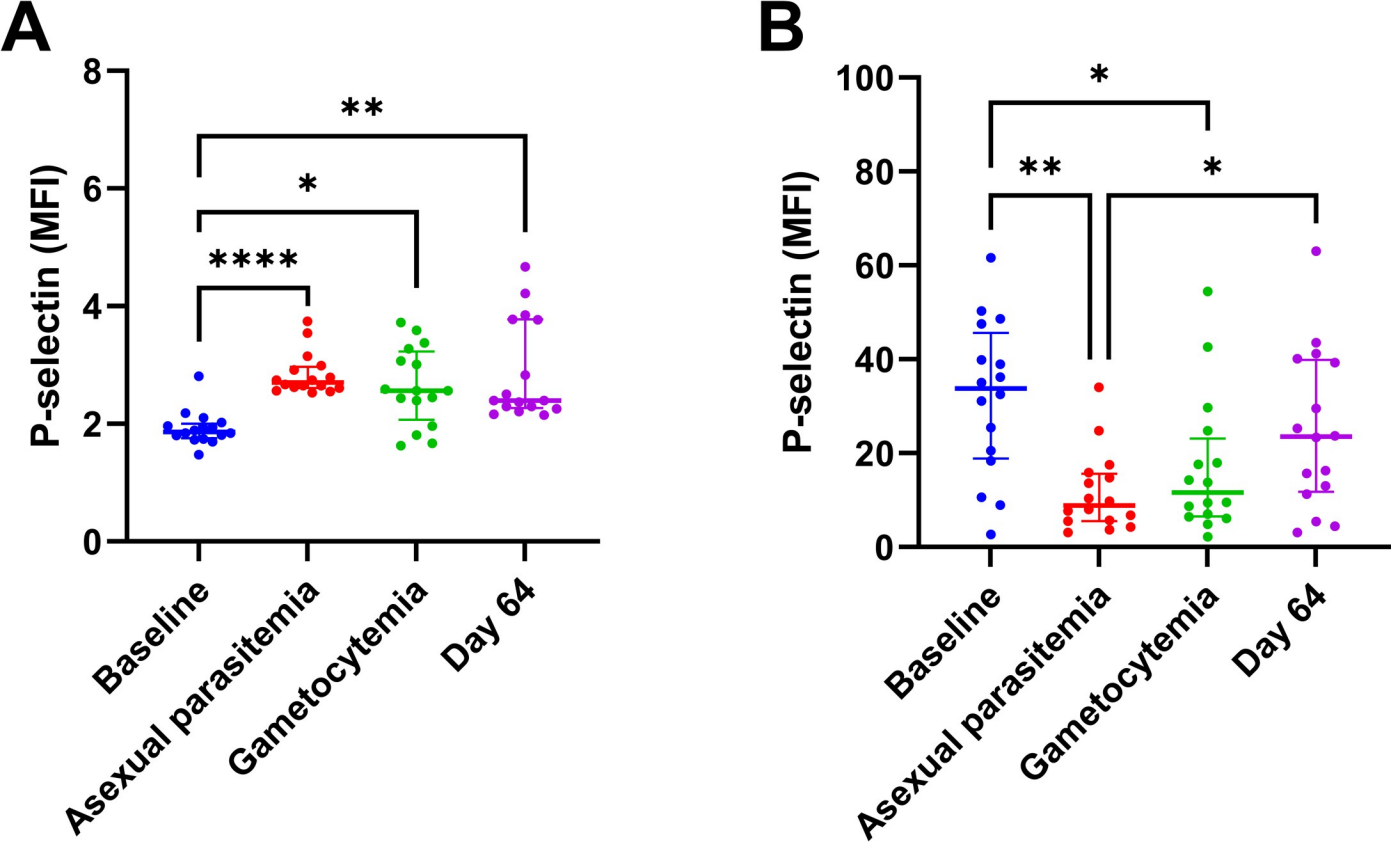
P-selectin expression in the four stages of *plasmodium falciparum* infection. (A) and platelets stimulated with CRP (B). (A) P-selection expression measured in unstimulated platelets was significantly increased at the time of asexual parasitemia, gametocytemia and convalescence compared to baseline. (B) P-selection expression measured in CRP-stimulated platelets was significantly decreased at the time of asexual parasitemia, gametocytemia and convalescence compared to baseline. Results are shown as the median and interquartile range, and *, **, and **** indicate p-values below 0.05, 0.01, and 0.0001, respectively.

We first investigated the effects of falciparum asexual parasitemia and gametocytemia on platelet number, activation status and reactivity. We found that platelet numbers were decreased at the peak of asexual parasitemia [182.6 (52.3) × 10^9^/L] compared to the baseline [306.7 (52.3) × 10^9^/L] and returned to normal during gametocytemia [306.7 (47.8) × 10^9^/L] and at day 64 after treatment [314.4 (57.0) × 10^9^/L] (Fig 1). We also calculated MPV over platelet count and found that the ratio MPV/platelet count was significantly increased (Fig 1). Flow cytometry was performed to study the activation status of circulating platelets, as well as their reactivity to *ex vivo* activation (Table 1). At the time of peak asexual parasitemia, P-selectin expression in unstimulated platelets was significantly increased, and this expression remained higher during gametocytemia and at day 64 compared to baseline (Fig 2). There were no differences in fibrinogen binding to the activated αIIbβ3 integrin in unstimulated platelets. There were also no significant changes in platelet reactivity to the different stimuli with the exception of a marked reduction in P-selectin and fibrinogen binding response to low concentrations of CRP-XL at the peak of asexual parasitemia and during gametocytemia.

**Table 1:** Platelet P-selectin expression and platelet-fibrinogen binding in response to ADP, CRP and TRAP in the four stages of *plasmodium falciparum* infection.

	Baseline	Asexual parasitemia	Gametocytemia	Day 64
**P-selectin (MFI)**				
Unstimulated	1.87 (1.76–2.00)	2.71 (2.61–2.97)****	2.56 (2.07–3.23)*	2.4 (2.27–3.79)**
ADP_high_	28.95 (20.69–34)	27.04 (17.53–31.09)	25.51 (17.09–31.29)	24.99 (17.72–29.54)
ADP_low_	12 (7.09–20.13)	11.83 (8.14–14.93)	11.6 (8.33–16.12)	12.39 (8.41–15.61)
CRP_high_	65.14 (60.2–70.75)	73.38 (66.67–76.43)*	67.11 (60.88–73.68)	68.25 (62.19–73.84)
CRP_low_	33.78 (18.90–45.65)	8.9 (5.55–15.61)**	11.67 (6.57–23.10)*	23.53 (11.77–39.9)
TRAP_high_	62.54 (54.02–71.75)	59.6 (51.22–69.99)	60.57 (57.99–64.60)	65.22(57.55–73.03)
TRAP_low_	5.37 (2.21–26.91)	4.79 (3.31–8.27)	4.28 (3.23–21.19)	5.26 (2.83–30.47)
**Fibrinogen binding (MFI)**				
Unstimulated	1.51 (1.42–1.54)	1.44 (1.38–1.50)	1.48 (1.31–1.50)	1.47 (1.42–1.54)
ADP_high_	11.93 (10.80–16.51)	11.94 (10.20–13.77)	9.93 (8.14–12.24)**	11.64 (10.24–13.84)
ADP_low_	6.01 (5.35–11.00)	6.2 (4.85–8.29)	5.08 (4.42–7.86)	7.33 (6.52–9.70)
CRP_high_	14.22 (11.61–16.54)	14.92 (12.69–16.56)	12.82 (11.01–15.74)	17.34 (14.99–21.31)**
CRP_low_	4.51 (3.23–7.52)	2.18 (1.82–3.19)**	2.46 (1.85–5.08)*	4.19 (2.93–8.66)
TRAP_high_	9.63 (8.48–14.80)	8.16 (7.35–11.66)	9.68 (6.93–11.60)	11.96 (10.27–14.04)
TRAP_low_	2.35 (1.65–3.99)	1.79 (1.53–3.11)	1.66 (1.40–4.96)**	1.84 (1.54–7.75)

Results at every stage are showed as median with IQR.

^*^P < 0.05

^**^P < 0.01

^***^P < 0.001; and

^****^P < 0.0001 according to Friedman or repeated measures ANOVA analysis, dependent on the distribution of the data.

### Von Willebrand factor and coagulation

**Fig 3:**
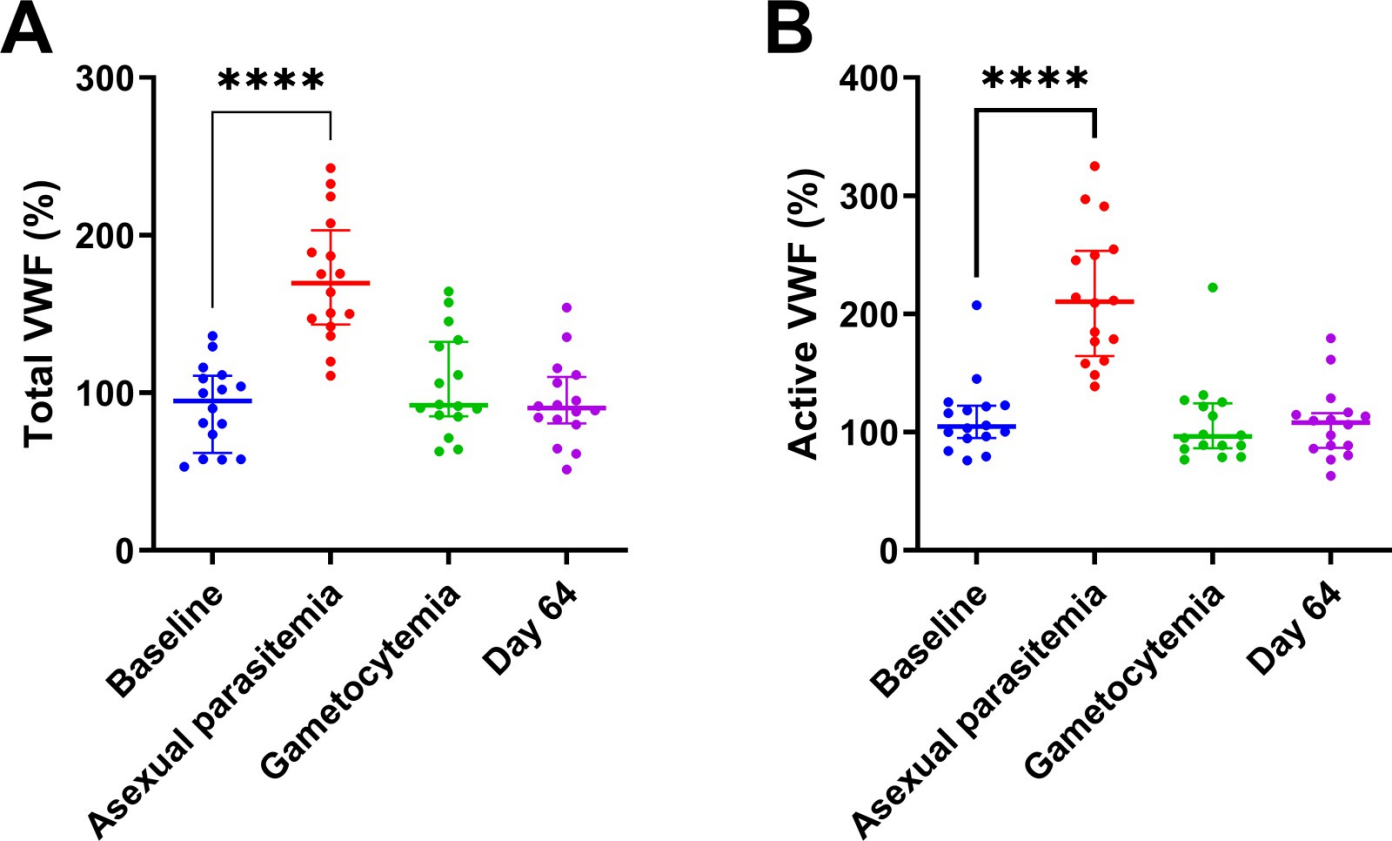
*Plasmodium falciparum* infection increases VWF and active VWF levels. **(A-B)** Total VWF (A) and active VWF (B) levels were quantified by ELISA and presented as the percentage of a pooled normal plasma standard. Results are shown as the median and interquartile range, and **** indicates a p-value below 0.0001.

To study the possible interaction between the observed changes in platelets with endothelial activation and coagulation, we measured plasma concentrations of VWF and active VWF and different markers of coagulation at the four timepoints. Fig 3 shows that VWF concentrations were significantly increased at the peak of asexual parasitemia [170% (143–203%); *P <* 0.0001], compared to baseline levels [95% (62–111%); *P =* 0.0037], but had returned to normal during gametocytemia [DT2; 92% (85–133%); *P <* 0.0001] and at day 64 [90% (81–110%); *P <* 0.0001]. Additionally, active VWF levels showed a similar pattern as VWF with higher values at peak asexual parasitemia [211% (165–253%)] compared to baseline [105% (95–123%), *P <* 0.0001], and returned to normal during gametocytemia [96% (87–125%), *P <* 0.001] and at day 64 [108% (87–116%); *P <* 0.0001] (Fig 3).

**Fig 4:**
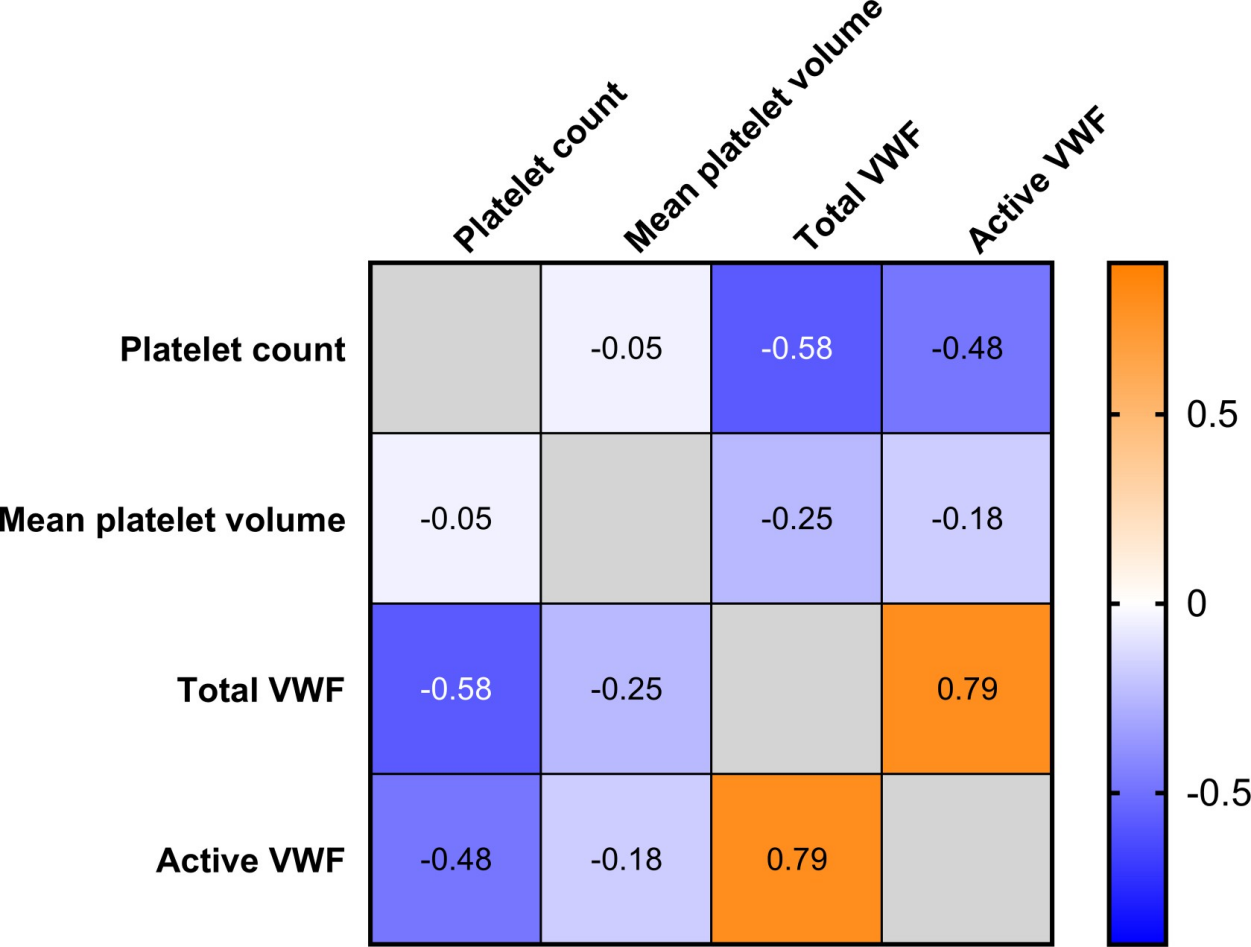
Correlation of platelet number and coagulation parameters. Correlation analysis shows that the platelet count is significantly associated with the total VWF level (p<0.001) and the active VWF level (P = 0.001). The mean platelet volume shows a tendency towards a correlation with active VWF levels, but this effect is statistically insignificant. (p = 0.093). Total and active VWF show a strong mutual association (p<0.001).

The levels of total and active VWF were negatively associated with the platelet count (p<0.001 for both), but were not significantly associated with the mean platelet volume (Fig 4).

Changes in coagulation were first assessed by measurement of plasma concentrations of prothrombin and fibrinogen and the anticoagulant factors antithrombin, α_2_Macroglobulin and protein S (Table 2). Overall, no significant changes in any of the factors were found at the peak of asexual parasitemia. The only significant changes were a small increase in the anticoagulant factors α_2_Macroglobulin during gametocytemia and antithrombin at day 64 (Table 2).

**Table 2:** Levels of pro- and anticoagulants in the four stages of plasmodium falciparum infection.

	Baseline	Asexual parasitemia	Gametocytemia	Day 64
**Prothrombin (%)**	100.0 (91.87–105.4)	96.26 (85.33–100.2)	91.24 (83.14–102.3)	95.98 (87.47–111.1)
**Fibrinogen (g/L)**	2.468 (2.008–2.924)	3.214 (2.215–4.698)	2.425 (1.989–4.191)	3.282 (2.881–3.851)
**Antithrombin (μM)**	105.7 (100.7–112.5)	107.7 (103.2–112.1)	109.5 (105.0–117.0)	113.8 (109.8–118.5)^**^
**α_2_M (μM)**	1.963 (1.607–2.313)	1.930 (1.585–2.286)	2.221 (1.785–2.513)^***^	1.990 (1.639–2.513)
**Protein S (%)**	100.0 (92.31–102.2)	94.26 (86.80–101.7)	101.4 (89.94–109.1)	97.44 (82.63–105.9)

Prothrombin and protein S levels were normalized to NPP. Differences between each group were determined using the Friedman Test with Dunn’s multiple comparison. All data are expressed median with IQR in parenthesis.

^**^P < 0.01 and ^***^P < 0.001 compared to baseline stage. α_2_M, α_2_Macroglobulin.

To further assess changes in coagulation, we performed a TG assay, which provides information on the overall coagulability (Table 3). The single difference at the time of peak asexual parasitemia was a small but significantly increase in TG lag time, which is the time needed to form the first traces of thrombin (P*<*0.0001). The ETP, which is the total amount of active thrombin formed and the peak height, which is the maximal amount of thrombin formed, were not increased at this time point. In contrast, both the ETP and peak were higher at the time of gametocytemia (ETP +15%; peak +21%), whereas the highest peak height value was observed at day 64 (Table 3). Thrombomodulin was added to the TG test to measure the reactivity of the activated protein C (APC) system. The thrombomodulin sensitivity of TG, quantified as the inhibition of the ETP, significantly increased during gametocytemia (+13%, p *=* 0.0156, Table 3). Overall, these findings suggest a slower onset of TG during asexual parasitemia, and a higher amount of thrombin formed during gametocytemia and at the time of convalescence, which is offset by increased sensitivity to the activities of protein C and the anticoagulants α_2_M and AT.

**Table 3:** Thrombin generation in the four stages of plasmodium falciparum infection.

	Baseline	Asexual parasitemia	Gametocytemia	Day 64
**Lag time (NPP%)**	100.0 (94.69–102.70)	119.5 (97.66–138.3)^****^	102.6 (89.21–117.0)	101.2 (86.67–102.6)
**ETP (NPP%)**	100.0 (82.12–124.8)	94.98 (90.72–112.8)	115.3 (94.54–133.8)^*^	108.8 (96.18–123.8)
**Peak (NPP%)**	100.0 (92.05–148.1)	120.7 (90.78–134.5)	121.2 (99.86–165.0)	134.4 (117.7–167.5)^*^
**Time to peak (NPP%)**	100.0 (93.20–118.6)	115.2 (91.12–135.6)	109.8 (98.43–143.5)^*^	101.7 (87. 83–126.1)
**VI (NPP%)**	100.0 (81.10–160.9)	115.0 (62.41–171.1)	124.5 (68.37–176.3)	140.5 (86.64–213.2)
**ETP inhibition by TM (%)**	52.39 (40.29–65.10)	62.60 (42.82–71.15)	59.44 (46.25–77.28)^*^	55.58 (42.09–71.61)

TG was measured at 5 pM TF. Differences between each group were determined by repeated measured ANOVA or Friedman test with Dunn’s correction, depending on the distribution of the data. TG parameters are expressed as a percentage of TG measured in pooled normal plasma. All data are expressed as median with IQR in parenthesis.

^*^P < 0.05 and ^****^P < 0.0001 compared to baseline stage.

**Fig 5:**
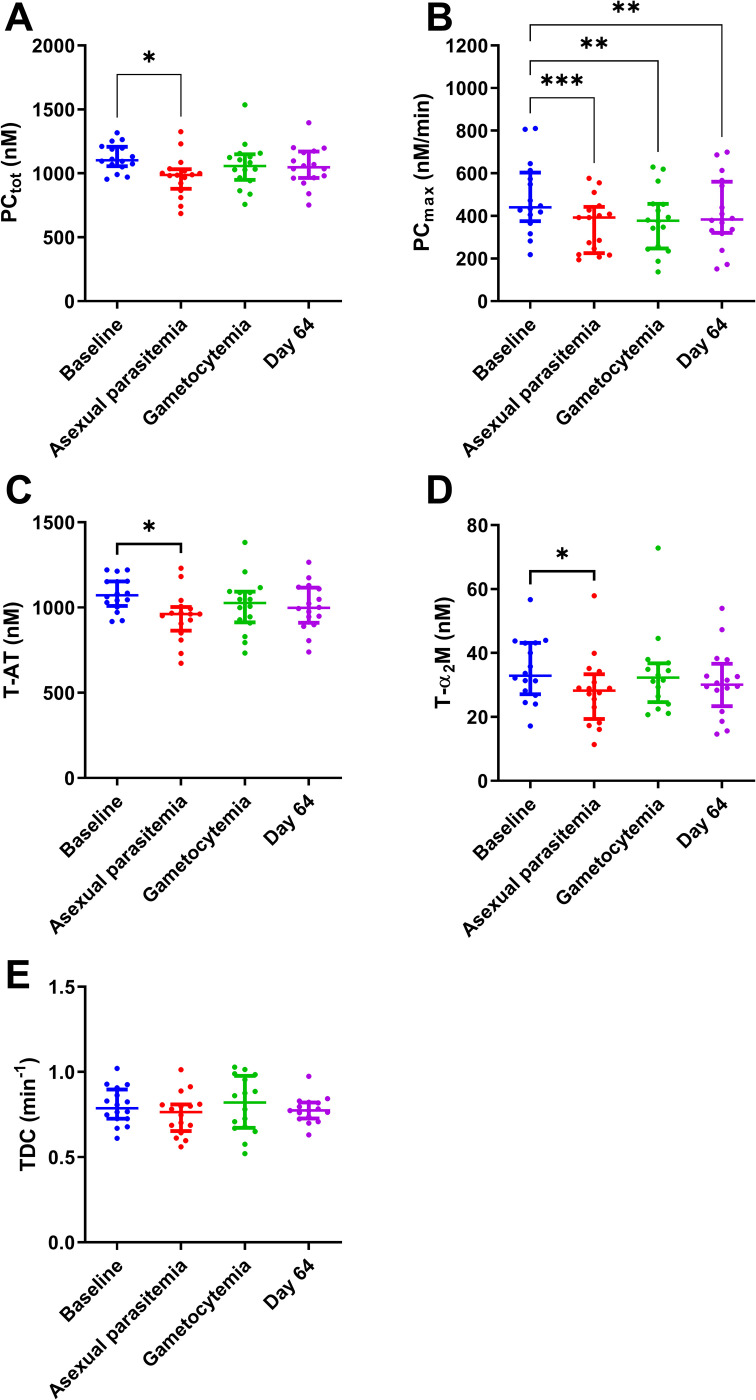
Thrombin dynamics analysis in the four stages of *plasmodium falciparum* infection. (A) The total amount of converted prothrombin(PC_tot_) is decreased during asexual parasitemia. (B) The maximum rate of prothrombin conversion (PC_max_) is decreased during asexual parasitemia. (C-D) Thrombin-antithrombin complex(T-AT) and Thrombin-α_2_M complex (T-α_2_M) formation are decreased during asexual parasitemia. (E) The thrombin decay capacity (TDC) remained unchanged. Results are shown as the median and interquartile range, and *, ** and *** indicate p-values below 0.05, 0.01, and 0.001, respectively.

To further investigate in depth the mechanism behind the changes in TG in malaria infection, we applied thrombin dynamics to quantify prothrombin conversion and thrombin inactivation (Fig 5). PC_tot_ and PC_max_ represented the total amount of converted prothrombin and the maximum rate of prothrombin conversion, respectively. Both PC_tot_ and PC_max_ were reduced at the peak of asexual parasitemia (-12.9%, *P =* 0.0185 and -26.1%, *P =* 0.0007). PC_tot_ was restored to its original level during gametocytemia and at day 64, whether PC_max_ remained lower at these time points (-22.3%, *P =* 0.0049 and -14.7%, *P =* 0.0078). Thrombin-antithrombin complex (T-AT) and thrombin-α2M complex (T-α2M) were reduced at moment of peak asexual parasitemia (-12.3%, *P =* 0.041 and -18.8%, *P =* 0.019). The thrombin decay capacity remained unchanged across time points.

**Fig 6:**
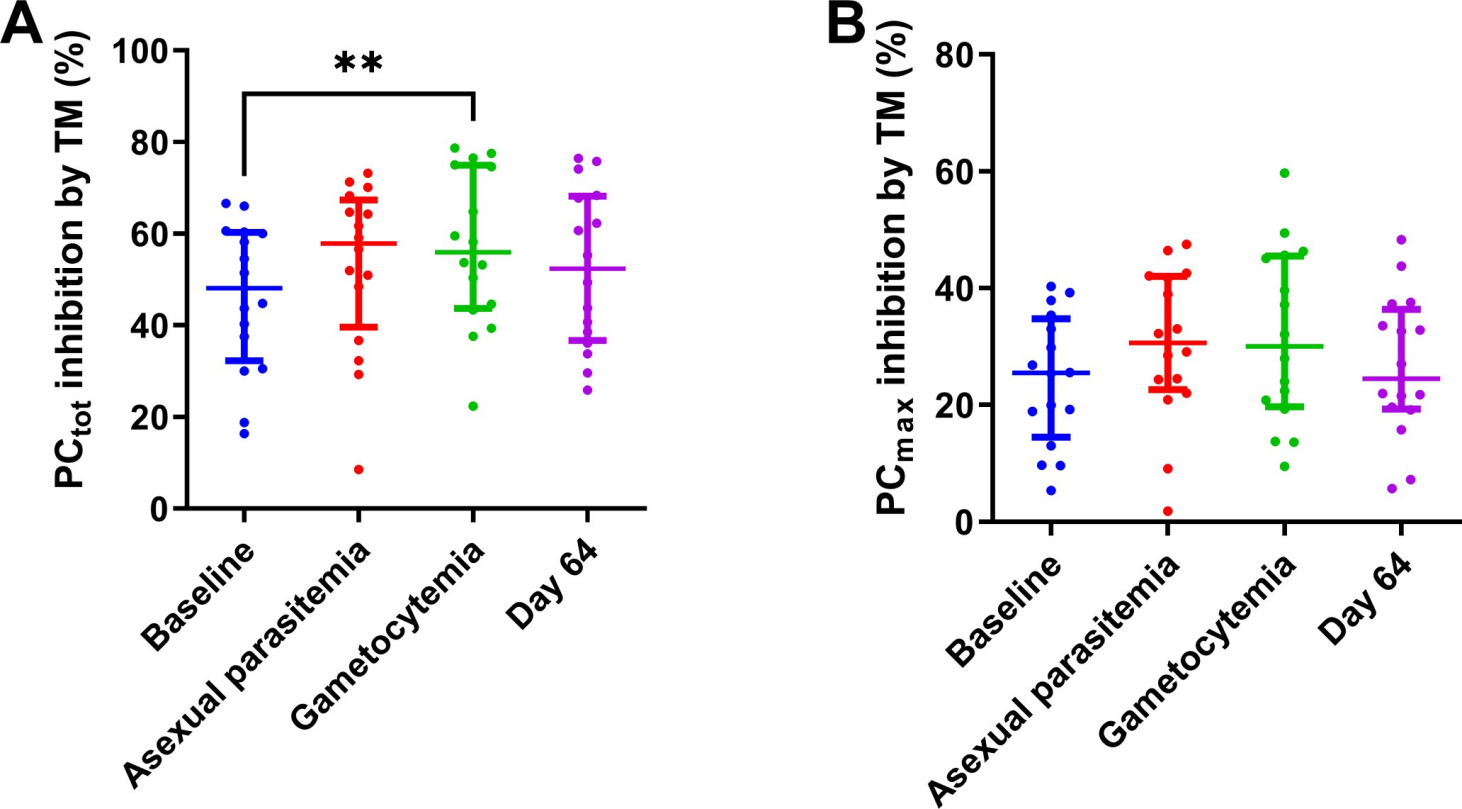
The inhibitory effect of thrombomodulin on prothrombin conversion in the four stages of plasmodium falciparum infection. (A) The thrombomodulin-induced inhibition of the total amount of converted prothrombin (PC_tot_; (A)) and the maximum rate of prothrombin conversion (PC_max_; (B)). Results are shown as the median and interquartile range, and ** indicates a p-value below 0.01.

The addition of thrombomodulin reduced PC_tot_ and PC_max_ by approximately 50% and 30% across all time points, and PC_tot_ was significantly stronger inhibited during gametocytemia than at baseline and the inhibition gradually decreased to normal after recovery (Fig 6).

### C-reactive protein

**Fig 7:**
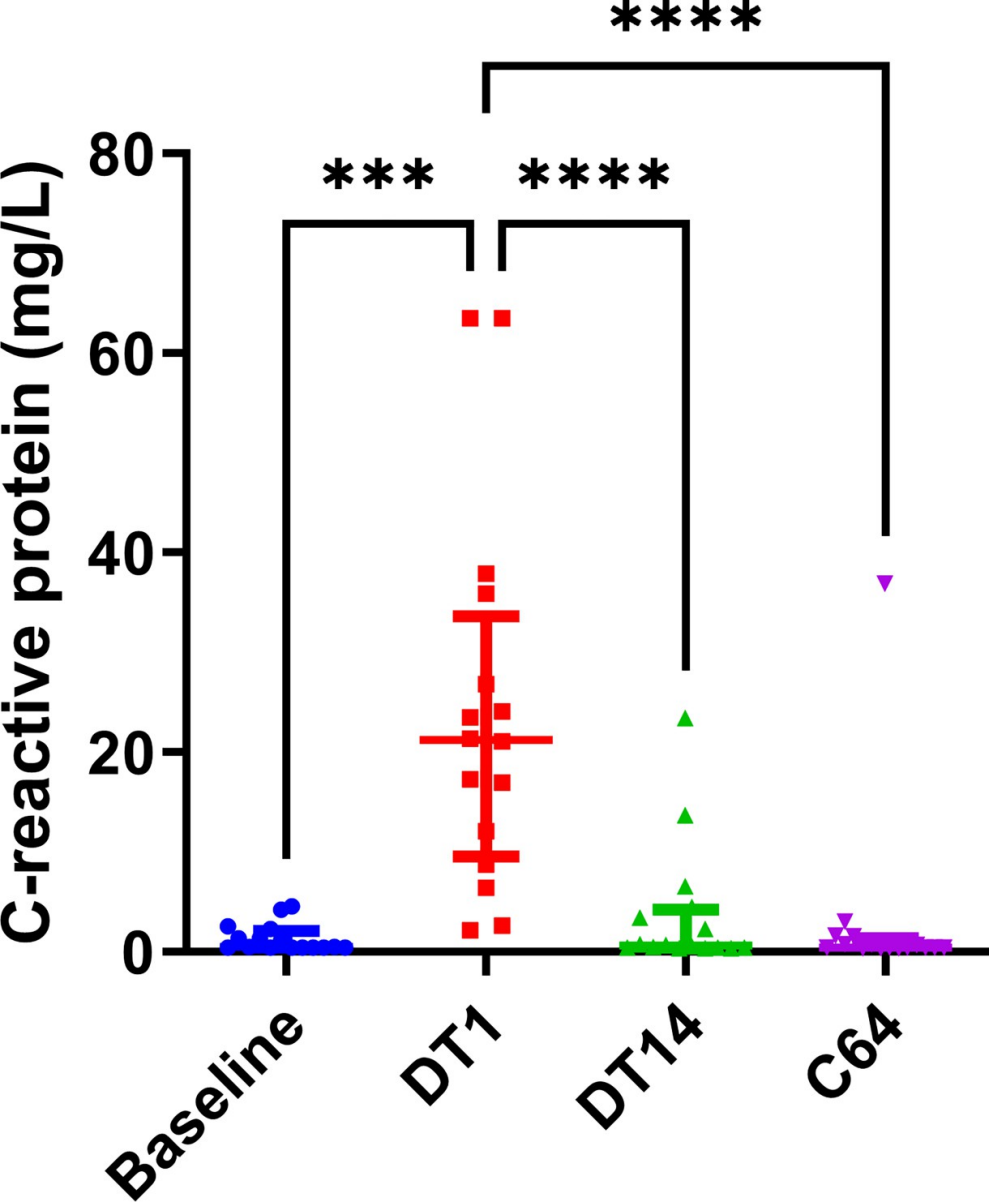
C-reactive protein levels in the four stages of plasmodium falciparum infection. Results are shown as the median and interquartile range, and *** and **** indicate a p-value below 0.001 and 0.0001, respectively.

Activation of platelets, coagulation and the endothelium are closely linked to inflammation. We therefore measured plasma concentrations of the inflammatory marker C-reactive protein (Fig 7). Our data shows that C-reactive protein concentrations were increased at the peak of asexual parasitemia compared to baseline (mean 24.0 mg/L ± 4.7 vs 1.3 mg/L ± 0.3), but returned to normal during gametocytemia (3.7 mg/L ± 1.6) and at day 64 (3.1 mg/L ± 2.3).

## Discussion

In this study, we assessed the functional changes in platelets induced by asexual and sexual *P*. *falciparum* parasitemia in a CHMI model and related these changes to coagulation and endothelial activation. We showed that platelet numbers decreased early during asexual parasitemia and that circulating platelets expressed more P-selectin, which is a sign of increased platelet degranulation, without an increased binding of fibrinogen to the activated αIIbβ3 integrin. This was not associated with platelet hyperreactivity, but, interestingly, platelets displayed a reduced P-selectin and fibrinogen binding response to stimulation with low dose XL-CRP during both asexual parasitemia and gametocytemia, suggesting a specific inhibition of collagen-mediated platelet activation. The mechanisms of this inhibition remain to be elucidated, but may include shedding of the collagen receptor (GPVI) by metalloproteinases of the A Disintegrin And Metalloproteinase (ADAM) family [26], as previously shown for GP1b in CHMI [16]. Overall, our findings confirm previous observations by our group that platelets are not or only mildly activated during early asexual parasitemia in CHMI and that platelet activation is unlikely to explain the early drop in platelet count [27].

Our findings also do not provide evidence that activation of coagulation, and more specific thrombin, underlies the decrease in platelet count [28–30]. We assessed activation of the coagulation by measuring plasma concentrations of different plasma pro- and anticoagulation markers, as well as assays of TG and thrombin dynamics [31, 32]. Only the lag-time showed a significant change during asexual parasitemia, with a trend towards a higher peak and an unchanged ETP. This is consistent with previous data from Riedl et al. [17], who reported an increase in thrombin peak during first detection of *P*. *falciparum* parasitemia in CHMI, without other changes in plasma markers of coagulation, including D-dimer and prothrombin fragment 1+2. In contrast, at the time of gametocytemia and convalescence, more thrombin was formed which resulted in the higher peak, ETP and velocity index. Nevertheless, decreased prothrombin levels, possibly in combination with reduced levels of other procoagulant factors cause the maximum rate of prothrombin conversion to be lower (PC_max_). However, this increase in thrombin activation was offset by increased sensitivity to the activities of protein C and higher plasma concentrations of the anticoagulants α_2_M and AT, suggesting a renewed balance between pro- and anticoagulation pathways.

In contrast to the limited changes in platelet reactivity and coagulation, plasma concentrations of total and active VWF were markedly increased at the moment of asexual parasitemia, which is in line with previous reports [33–35]. Under physiological conditions, VWF is essential to recruit platelets from the circulation to injury sites, where they act to prevent excessive bleeding [36]. Active VWF is VWF in a conformation that can spontaneously bind platelets and our present findings support the notion that endothelial activation, resulting in the secretion of ultra-large VWF plays a key role in the disease process by binding both platelets and infected red cells [33, 35]. Early endothelial activation is of paramount importance for the malaria parasite as it facilitates sequestration of iRBC, thereby preventing their removal by the spleen. Moreover, in the circulation, VWF is the carrier of coagulation factor FVIII, protecting it from degradation and extending its half life [36].

More recently, a novel role for VWF has been proposed as a mediator of crosstalk between the coagulation system and immune cells, such as neutrophils, macrophages and leukocytes [36]. We recently showed that CHMI induced long-term functional changes in monocytes [13]. We here showed that changes in platelet P-selectin expression, their reactivity to low dose CRP-XL and changes in coagulation persist for longer time as well. In contrast, the increase in VWF and active VWF was short lived as levels had returned to baseline at the time of gametocytemia. The observation that the changes in platelets and coagulation persisted until day 64 after challenge, suggest that these changes represent a temporal adaptation to a recent inflammatory process rather than a separate effect of gametocytemia. Whether these long-term changes are more outspoken in naturally-acquired, more severe malaria infections remains to be established.

## Supporting information

S1 Dataset (XLSX)
